# Prospective observational study on the relationships between genetic alterations and survival in Japanese patients with metastatic castration-sensitive prostate cancer: the impact of IDC-P

**DOI:** 10.1007/s10147-025-02707-3

**Published:** 2025-02-12

**Authors:** Masashi Kato, Hiroyuki Sato, Yushi Naito, Akiyuki Yamamoto, Hideji Kawanishi, Yojiro Nakano, Toshinori Nishikimi, Masataka Kobayashi, Atsuya Kondo, Hiroki Hirabayashi, Satoshi Katsuno, Fumitoshi Sakamoto, Tohru Kimura, Shigeki Yamamoto, Hidemori Araki, Kosuke Tochigi, Fumihiro Ito, Hatsuro Hatsuse, Naoto Sassa, Akihiro Hirakawa, Shusuke Akamatsu, Toyonori Tsuzuki

**Affiliations:** 1 Department of Urology, Japanese Red Cross Aichi Medical Center Nagoya Daiichi Hospital, 3-35 Michishita-Cho, Nakamura-Ku, Nagoya, 453-8511 Japan; 2 Division of Biostatistics and Data Science, Clinical Research Center, Tokyo Medical and Dental University, Tokyo, Japan; 3 Department of Urology, Nagoya University Graduate School of Medicine, Nagoya, Japan; 4 Department of Urology, Toyohashi Municipal Hospital, Toyohashi, Japan; 5 Department of Urology, Nagoya Medical Center, Nagoya, Japan; 6 Department of Urology, Tosei General Hospital, Seto, Japan; 7 Department of Urology, Japanese Red Cross Aichi Medical Center Nagoya Daini Hospital, Nagoya, Japan; 8 Department of Urology, Narita Memorial Hospital, Toyohashi, Japan; 9 Department of Urology, Kariya Toyota General Hospital, Kariya, Japan; 10 Department of Urology, Okazaki City Hospital, Okazaki, Japan; 11 Department of Urology, Chubu Rosai Hospital, Nagoya, Japan; 12 Department of Urology, Japan Community Healthcare Organization Chukyo Hospital, Nagoya, Japan; 13 Department of Urology, Tsushima City Hospital, Tsushima, Japan; 14 Department of Urology, Meitetsu Hospital, Nagoya, Japan; 15 Department of Urology, Yokkaichi Municipal Hospital, Yokkaichi, Japan; 16 Department of Urology, Gifu Prefectural Tajimi Hospital, Tajimi, Japan; 17 Department of Urology, Ichinomiya Municipal Hospital, Ichinomiya, Japan; 18 Department of Urology, Aichi Medical University, Nagakute, Japan; 19 Department of Surgical Pathology, Aichi Medical University, 1-1 Yazakokarimata, Nagakute, Aichi 480-1195 Japan

**Keywords:** Metastatic castration-sensitive prostate cancer (mCSPC), Genetic alterations, BRCA, Intraductal carcinoma of the prostate (IDC-P), Cancer-specific survival, Homologous recombination repair gene mutation (HRRm)

## Abstract

**Background:**

Intraductal Carcinoma of the Prostate (IDC-P) is a significant prognostic indicator for prostate cancer, which demonstrates significant associations with homologous recombination repair gene mutations (HRRm) and alterations in tumor suppressor genes. However, no study in Japan has investigated the association between IDC-P and genetic mutations in men with metastatic castration-sensitive prostate cancer (mCSPC).

**Methods:**

This prospective observational study enrolled 102 de novo mCSPC (LATITUDE high-risk) patients diagnosed between 2018 and 2021, with subsequent monitoring of survival outcomes. A single genitourinary pathologist evaluated all needle biopsy slides. Genetic analyses were performed using the Myriad myChoice HRD plus™. These genetic analyses covered 108 genetic loci, including 15 HRRm genes, with a success rate of 91%.

**Results:**

Genetic alterations were observed in 79 patients (77.5%), with 20 exhibiting HRRm (19.6%). Common genetic alterations included *FOXA1* (29.4%) and *TP53* (17.6%) mutations; *BRCA* (9.8%) mutations were the most frequent HRRm (*BRCA1*:2 cases, *BRCA2*:8 cases, including 6 biallelic). IDC-P-positive patients demonstrated a significantly higher frequency of genetic aberrations (82.6% vs. 50%, *p* = 0.0082). Patients with biallelic *BRCA2*, *TP53*, and *PTEN* mutations exhibited significantly poorer cancer-specific survival. Multivariate analysis identified lactate dehydrogenase (LDH) (HR 1.005, *p* = 0.035), TP53 mutations (HR 5.196, *p* < 0.001), biallelic BRCA2 mutations (HR 10.686, *p* = 0.005), and IDC-P as independent predictors of poor cancer-specific survival. No cancer-related deaths occurred in IDC-P-negative cases.

**Conclusion:**

Our study emphasizes the significant association between IDC-P and an elevated incidence of genetic alterations in Japanese mCSPC patients, emphasizing the need for early genetic testing to guide therapeutic decision-making.

**Supplementary Information:**

The online version contains supplementary material available at 10.1007/s10147-025-02707-3.

## Introduction

Intraductal carcinoma of the prostate (IDC-P) is a high-grade, high-volume, invasive prostate cancer (PCa) with poor clinical outcomes; it manifests as well-circumscribed lesions with intact basal cells lining distended ducts infiltrated by malignant epithelium [1, 2]. Its development and aggressiveness are due to a series of genomic and epigenomic alterations in the prostate gland during tumorigenesis [3]. The International Society of Urological Pathologists (ISUP) recommends incorporating IDC-P into the Gleason score [4]. This poor prognostic factor is associated with germline *BRCA2* mutations, other homologous recombination repair gene mutation (HRRm), and tumor suppressor genes alterations [3, 5]. Risbridger et al. demonstrated for the first time an increased presence of IDC-P in patient-derived xenografts model from g*BRCA2* mutation carriers [6]. Similarly, Böttcher et al. revealed an association between the presence of IDC-P in ≥ 30% of tumor and genomic instability [7]. In addition, a recent study identified DNA repair defects in cell-free plasma DNA from one-third of IDC-P patients, consistent with findings in somatic tissue [8]. Notably, *PTEN* loss and *ERG* expression are frequently observed in IDC-P, whereas both are uncommon in high‐grade prostatic intraepithelial neoplasia (HGPIN) [9].

National comprehensive Cancer Network (NCCN) and European Association of Urology (EAU) guidelines recommended genetic testing in patients with IDC-P and cribriform hjstology [10, 11]. The recent guidelines have weakened recommendations for genetic testing in intermediate-risk prostate cancer with IDC-P, but testing is recommended in metastatic castration-sensitive prostate cancer (mCSPC) patients [12]. However, a recent study reported no association between IDC-P or cribriform histology and germline *BRCA2* mutation [13], indicating the relationship between IDC-P and germline *BRCA2* mutations remains ambiguous. Additionally, these data on survival outcomes for mCSPC patients often rely on retrospective analyses.

To address these gaps, we initiated a prospective observational multi-institutional study. This study aims to prospectively analyze the characteristics of genetic mutations, including *BRCA2* mutations, in Japanese mCSPC patients with and without IDC-P. Furthermore, we prospectively followed and analyzed patient survival outcomes using real-world data.

## Patients and methods

### Study design and ethics statement

Between 2018 and 2021, we prospectively enrolled 102 de novo mCSPC patients at Nagoya University and affiliated healthcare facilities. Inclusion criteria were as follows: Patients diagnosed with mCSPC based on prostate needle biopsies and classified as high-risk according to the Latitude criteria (meeting at least two of the following three factors: Gleason score ≥ 8, ≥ 3 lesions in bone scan, and the presence of measurable visceral lesions in computed tomographic, bone scintigraphy, or magnetic resonance imaging) [14]. Additionally, patients who underwent a standard extended biopsy ≥ 10 cores to ensure a sufficient tumor tissue for genetic testing were included. After screening 116 patients, genetic mutation data were available for 106 patients. Finally, 102 met the inclusion criteria and had available genetic mutation data. Four individuals were excluded due to insufficient metastatic lesions or inadequate biopsy specimens. The Ethics Committee of Nagoya University and each institution approved the study protocol (2020–0067 19940). Informed consent was obtained from all study participants. This study was conducted in accordance with the Declaration of Helsinki.

A single genitourinary pathologist evaluated all needle biopsy slides in accordance with the 2019 International Society of Urological Pathology (ISUP) grading system. Genetic analyses were performed using Myriad Genetics Inc., encompassing a comprehensive assessment of 108 genes through the application of the Myriad myChoice HRD plus™ assay (Myriad Genetics Inc., Salt Lake City, UT, USA) to assess 108 genes comprehensively; this assay detects sequence variants and large rearrangements in 15 HRR-related genes, including *BRCA1, BRCA2, ATM, BARD1, BRIP1, CDK12, CHEK1, CHEK2, FANCL, PALB2, PPP2R2A, RAD51B, RAD51C, RAD51D*, and *RAD54L*.

### Statistical analysis

The prevalence of genomic mutations was compared between IDC-P-positive and -negative groups using the Chi-square test or Cochran–Mantel–Haenszel (CMH) test, with stratification factors in the CHM test chosen based on literature review and medical clinical expertise. All statistical tests were two-sided with a significance level of 5%. Point estimate and their corresponding 95% Clopper–Pearson confidence intervals (Cis) were calculated for each group. To ensure complete data for the primary analysis, efforts were made to obtain gene analysis results for all cases. In addition, we summarized the mutation frequencies of individual HRR-related genes stratified by IDC-P status.

The Kaplan–Meier method was used to estimate the probability of event-free survival The stratified log-rank test was used to assess differences in survival curves between IDC-P-positive and -negative groups. To identify independent predictors of survival, a multivariable Cox proportional hazards regression model was used to evaluate hazard ratios (HRs) and the 95% Cis for IDC-P status and other included in the stratified log-rank test.

For cancer-specific survival with competing risk of death from any causes, we compared cumulative incidence curves between IDC-P-positive and -negative groups using Gray’s test. A similar approach was used to compare HRRm-positive and -negative groups. Fine and Gray’s competing risks model with adjustment for relevant prognostic factors was used to estimate HRs and 95% Cis for these comparisons.

## Results

### Baseline patient characteristics

Table 1 presents the baseline characteristics of the study participants. The median age was 72 years (57–92), and the median serum level of PSA was 441 ng/ml (3.9–9807) ng/ml. The median follow-up period was 38.3 (8.2–67.4 months) months. All patients had a Gleason grade > 3, with 66 (64.7%) exhibiting Gleason pattern 5. We identified lymph node metastasis in 67 patients (65.7%) and visceral metastasis in 23 (22.5%; lung [*n* = 22], liver [*n* = 1], pancreas [*n* = 1], and meninges [*n* = 1]); bone metastasis was observed in all patients. We achieved a 91% success rate for genomic testing. Finally, 102 de novo mCSPC (LATITUDE high-risk) patients were included. IDC-P was identified in 86 (84%) patients; no significant differences were observed in patient characteristics between IDC-P -positive and -negative cases (Table 1). Biopsy Gleason score tended to be higher in IDC-P positive patients.

**Table 1:** Clinicopathological characteristic of the 102 patients with metastatic hormone-sensitive prostate cancer

	IDC-P Negative	IDC-P Positive	*P* value*
	16 cases	86 cases	
Age			
Median(25%ile, 75%ile)	74 (66,77.5)	71 (67,77)	0.59
Min, max	62,87	57,92	
Gleason score			0.08
4 + 3	1 (6.3%)	1 (1.2%)	
4 + 4	6 (37.5%)	28 (32.6%)	
4 + 5	5 (31.3%)	25 (29.1%)	
5 + 3	2 (12.5%)	1 (1.2%)	
5 + 4	2 (12.5%)	22 (25.6%)	
5 + 5	0	9 (10.5%)	
iPSA (ng/mL)			0.47
Mean ± SD	1547.2 ± 1806.5	1180.7 ± 1852.4	
Median(25%ile, 75%ile)	912 (135.8,2515)	356 (118.3,1158)	
Min, max	26.8, 5380.0	3.9, 9807.0	
ECOG PS			0.24
0	13 (81.25%)	55 (64.71%)	
1	2 (12.50%)	23 (27.06%)	
2	0	4 (4.71%)	
3	0	3 (3.53%)	
4	1 (6.25%)	0	
cN			0.78
N0	6 (37.50%)	28 (32.94%)	
N1	10 (62.50%)	57 (67.06%)	
Bone metastasis			0.11
1 ~ 2	0	4 (4.76%)	
3 ~ 5	2 (12.50%)	16 (19.05%)	
6 ~ 20	4 (25.00%)	26 (30.95%)	
≥ 20	7 (43.75%)	32 (38.10%)	
Super Scan	3 (18.75%)	6 (7.14%)	
Visceral metastasis			0.76
(-)	13 (81.25%)	65 (76.47%)	
( +)	3 (18.75%)	20 (23.52%)	
The frequency of genetic alterations			0.008
(-)	8 (50.00%)	15 (17.4%)	
( +)	8 (50.00%)	71(82.6%)	

### Genetic alterations

Analysis of genetic alterations revealed mutations in 79 patients (77.5%). HRRms were identified in 20 patients (19.6%), with *BRCA*【*BRCA1*: 2 cases, *BRCA2*:8 (monoallelic 2, biallelic 6) 】, followed by *CDK12* mutations (*n* = 6). The most frequent genetic alterations were *FOXA1* (29.4%) and *TP53* (17.6%) mutations. The frequency of genetic alterations was significantly higher in IDC-P -positive patients than in IDC-P -negative patients (82.6% vs. 50%, *p* = 0.0082; Table 1). Furthermore, *FOXA1*, *PTEN,* and *TP53* mutations were frequent in IDC-P-positive patients (*FOXA1*: 31.4% vs. 18.8%, *PTEN*: 5.8% vs. 0%, *TP53*: 20.9% vs. 0%, *p* = 0.019, 1, and 0.068). No significant difference was observed in *BRCA1/BRCA2*, *CDK12*, or *ATM* mutations between the groups (Supplement 1). *PTEN* mutations were detected exclusively in tumors with IDC-P and Gleason pattern 5. *TP53* mutations were identified only in IDC-P-positive cases. Specifically, the *TP53* mutation-positive ratio was 2/29 in cases without Gleason pattern 5 and 16/57 in cases with Gleason pattern 5 (*p* = 0.51).

### Prognostic factors for cancer-specific survival (CSS)

**Fig. 1:**
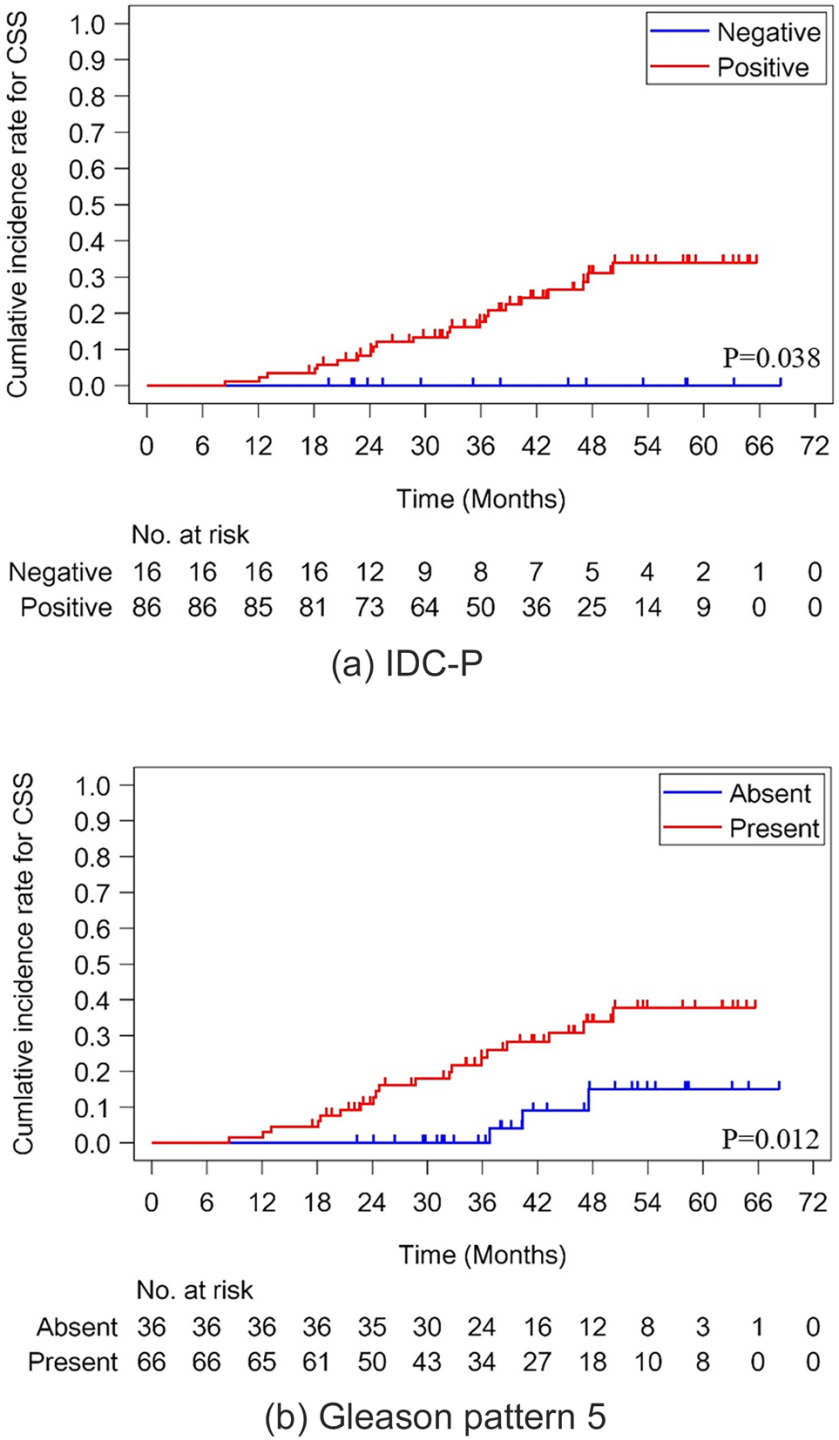
Prognostic impact of pathological and genetic features in metastatic castration-sensitive prostate cancer (mCSPC) patients. **a** Association between cancer-specific survival and intraductal carcinoma of the prostate (IDC-P). **b** Association between cancer-specific survival and Gleason pattern 5

**Fig. 2:**
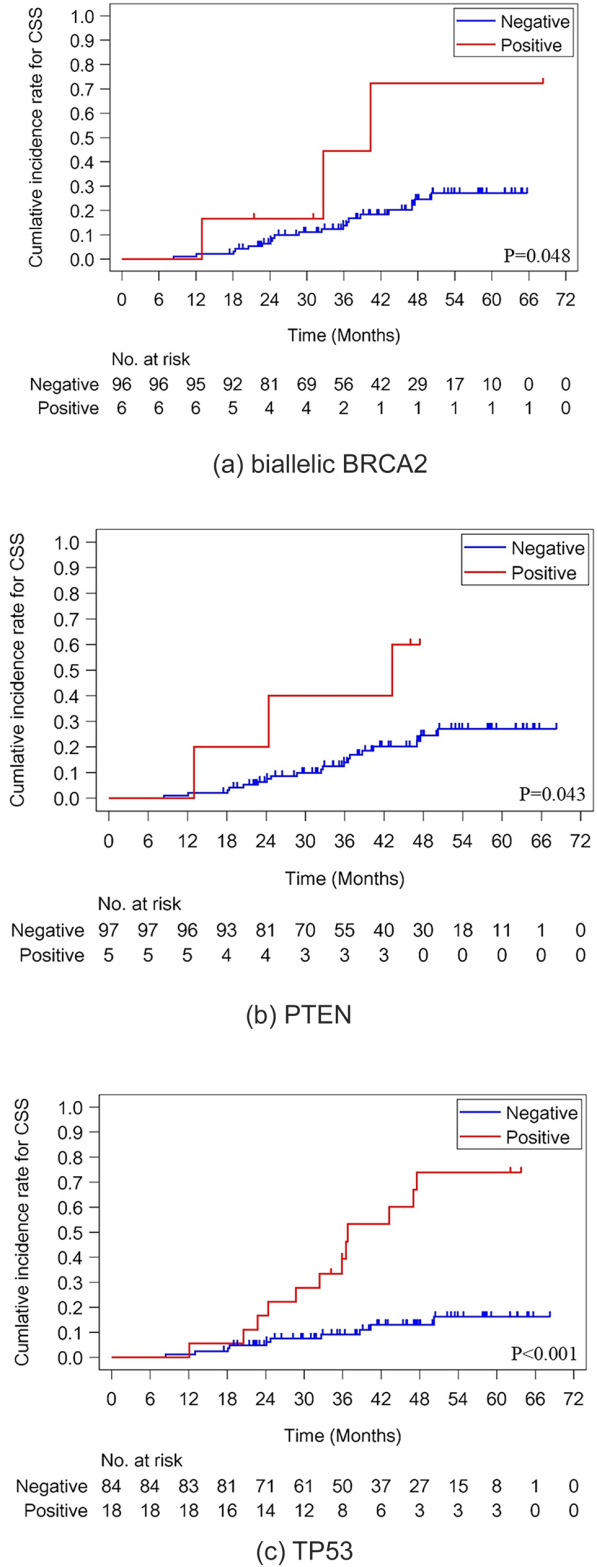
Prognostic impact of pathological and genetic features in metastatic castration-sensitive prostate cancer (mCSPC) patients. **a** Association between cancer-specific survival and biallelic *BRCA2* mutations. **b** Association between cancer-specific survival and *PTEN* loss. **c** Association between cancer-specific survival and *TP53* mutations

In total, 27 patients died during follow-up (22 from the disease and 5 from other causes). Gleason pattern 5 (*p* = 0.012) and IDC-P (*p* = 0.038) were significant negative prognostic factors for CSS by Gray’s test (Fig. 1a, b). In addition, patients with biallelic mutations in *BRCA2*, *PTEN*, and *TP53* exhibited significantly shorter CSS compared to those without mutations by Gray’s test (*p* = 0.048, *p* = 0.043, and *p* < 0.0001, respectively; Fig. 2a–c). On the other hand, no significant difference was observed in *BRCA1/BRCA2*. Multivariate analysis identified lactate dehydrogenase (LDH) (HR 1.005, *p* = 0.035), *TP53* mutations (HR 5.196, *p* < 0.001), biallelic *BRCA2* mutations (HR 10.686, *p* = 0.005), and IDC-P as independent predictors of poor CSS (Table 2). Notably, the HR could not be calculated in IDC-P-negative cases due to the absence of cancer-related death during follow-up period.

**Table 2:** Cancer-specific survival (102 cases) and variables (Fine and Gray model)

Variables	Comparison	Univariate analysis		Multiparametric analysis	
		HR [95% CI]	*P* value	HR [95% CI]	*P* value
IDC-P	Presence vs. Absence	Immposible to estimate***		Immposible to estimate***	
Gleason pattern 5	Presence vs. Absence	3.640 [1.312, 13.744]	0.0296	2.154 [0.629, 9.504]	0.2690
HRRm	Presence vs. Absence	1.376 [0.476, 3.378]	0.5240		
Genetic alterations	Presence vs. Absence	1.554 [0.561, 5.867]	0.4577		
*BRCA*	Presence vs. Absence	1.959 [0.518, 5.447]	0.2583		
bi-allelic *BRCA2*	Presence vs. Absence	4.081 [1.076, 11.394]	0.0185	10.686 [1.769, 50.462]	0.0049
*CDK12*	Presence vs. Absence	2.243 [0.449, 7.046]	0.2408		
*TP53*	Presence vs. Absence	6.224 [2.729, 14.480]	< .0001	5.196 [2.074, 14.001]	0.0009
*PTEN*	Presence vs. Absence	4.168 [1.094, 11.760]	0.0177	2.490 [0.609, 8.022]	0.1752
iPSA	Continuous variables	1.205 [0.519, 3.060]	0.6827		
cN	N1 vs. N0	0.531 [0.141, 1.471]	0.2860		
cM	1c vs. 1b	0.470 [0.124, 1.305]	0.2041		
Viceral metasitasis	Presence vs. Absence	1.000 [1.000, 1.000]	0.4226		
EOD	Continuous variables	1.110 [0.745, 1.674]	0.6137		
Hb	Continuous variables	0.909 [0.762, 1.107]	0.3185		
ALP	Continuous variables	1.000 [1.000, 1.001]	0.1293		
LDH	Continuous variables	1.005 [1.001, 1.009]	0.0113	1.005 [1.000, 1.009]	0.0347

*HRRm* homologous recombination repair gene mutation, *EOD* extent of disease, *ALP* alkaline phosphatase, *LDH* lactate dehydrogenase

*There was no event of cancer death in IDC-P negative cases

## Discussion

We identified a significant association between IDC-P, specific genetic mutations, and poor CSS in Japanese patients with mCSPC. Notably, Gleason pattern 5, IDC-P, and biallelic mutations in *BRCA2*, *TP53*, and *PTEN* were identified as significant adverse prognostic factors. Furthermore, multivariate analysis revealed that biallelic mutations in *BRCA2*, *TP53*, LDH, and IDC-P were significantly associated with worse CSS.

ZENSHIN, a Japanese observational study, reported that approximately one-third of Japanese patients with metastatic castration-resistant prostate cancer (mCRPC) carry HRRm, [15] in contrast to our study. This difference may be attributable to the fact that the patients analyzed in mCRPC includes more advanced and aggressive cancers compared to mCSPC. Although the ZENSHIN study retrospectively analyzed clinical outcome, definitive conclusions regarding survival were not established. Prognostic evaluations of PCa incorporating genetic mutations in Japanese patients have largely relied on retrospective studies, [15, 16] emphasizing the importance of our prospective study. In Japan, genetic testing is exclusively permitted for patients with advanced mCRPC under health-insurance coverage. Consequently, the dataset generated from our study, conducted at the time of mCSPC diagnosis, holds significant value.

*BRCA2* mutation carriers exhibit an increased lifetime risk of developing PCa and a poorer prognosis than noncarriers [17]. Moreover, *BRCA-2* mutant tumors commonly harbor concurrent IDC-P pathology [18]. Furthermore, *BRCA2-*mutant PCa harboring IDC-P is associated with genomic and epigenomic dysregulation of the *MED12L/MED12* axis [18]. Several studies have demonstrated an association between IDC-P and germline *BRCA2* mutations [5, 8]. A recent study from Spain reported no association between IDC-P and germline *BRCA2* mutations; however, significant associations were observed between IDC-P and biallelic *BRCA2* loss and *PTEN* homozygous loss in primary tumors [19], consistent with our findings. In addition, responses to poly-adenosine diphosphate-ribose polymerase inhibitors (PARPi) have been associated with homozygous *BRCA2* somatic loss in the primary tumor [20]. The relationship between IDC-P and germline *BRCA2* mutations remains uncertain.

However, several studies have revealed significant association between IDC-P and *TP53, RB1*, and *PTEN* [21]. Cribriform and IDC-P show a higher percentage of genome alterations and somatic copy number alterations, including loss of *PTEN*, gain of *MYC*, and point mutation of *TP53* [4]. Recent studies have emphasized that *PTEN* is significantly associated with IDC-P and poor patient outcomes [22]. Furthermore *TP53, PTEN*, *RB1* loss, and *MYC* amplification are reported to be associated with resistance to hormonal therapy, and these genetic alterations are frequently present in IDC-P-positive mCSPC patients; [19] which is consistent with our data. A study that employed a clinically validated genomic test demonstrated that the IDC-P or cribriform is significantly associated with aggressive signatures [23]. In this study, cribriform status or IDC-P was significantly associated with elevated median Decipher scores and Decipher risk category. Similarly, we found that IDC-P-positive patients had significantly more genetic alterations in primary tumors. Moreover, we conducted the significant prognostic value of *TP53* mutations*,* biallelic mutations in *BRCA2*, and IDC-P. Originally, TP53 and BRCA2 mutations have been associated with IDC-P. However, multivariate analysis in our study has demonstrated that IDC-P is a significant factor alongside these genetic abnormalities. This suggests that IDC-P may encompass factors beyond those explained by these genetic mutations alone, warranting further investigation in future studies. Wilkinson et al. identified *TP53* alterations, *PTEN* loss, nuclear *ERG* expression, tumor volume, and IDC-P as potential predictors of response to androgen deprivation therapy, with an area under the curve of 0.98 [24]; this implies a potential role for IDC-P as a biomarker for treatment efficacy.

In this study, the success rate of genetic testing was over 90% in Japanese mCSPC patients with prostate biopsy samples within 3 years of sampling. This is significantly higher than the rates reported in previous large-scale studies, including PROfound (69%), TRITON2 (68%), and ZENSHIN (72%) [15, 25, 26]. Several factors may have contributed to these discrepancies. First, we included patients classified as high-risk based on LATITUDE criteria, leading to larger and more easily sampled tumor. Second, we used fresh samples (within 3 years), improving DNA quality. Finally, all patients underwent systematic needle biopsy with ≥ 10 core extended biopsies, regardless of mCSPC status. Although a lower number of needle biopsy core is preferred for men with mCSPC compared to those with early-stage disease, this approach can lead to cores with low volume or low tumor cellularity due to the inherent limitations of blind needle biopsy. Consequently, a reduced number of cores may compromise the success rate of genetic testing. Furthermore, we previously reported a positive correlation between the number of cores obtained through systemic biopsy and the detection rate of IDC-P, leading to more precise patient management [27]. This observed association warrants further investigation, as it has the potential to significantly improve testing protocols. Therefore, we recommend a systematic needle biopsy approach even in metastatic cases.

Our study had several limitations. First, the sample size was relatively small. Second, due to the observational nature of the study, treatment regimens with novel androgen receptor signaling inhibitors and chemotherapy varied among participants. Third, the inclusion criteria for LATITUDE high-risk disease resulted in more patients with IDC-P. As we previously reported that the IDC‐P‐positivity rate was 66.7% in patients with de novo metastatic disease included low burden [3], this positivity rate was not far off. Conversely, this elevated positivity rate may complicate the differentiation between IDC-P-positive and -negative cases within the study, specifically those with HRRm, including *BRCA* mutations. Fourth, the current lack of a precise definition for IDC-P fosters confusion surrounding its diagnosis. It is imperative that consensus criteria for IDC-P be established to enhance diagnostic consistency and propel research forward in prostate cancer. Such consensus will enable more accurate and comparable data across studies, which is essential for improving patient outcomes.

In conclusion, we identified frequent mutations in *BRCA*, *FOXA1*, and *TP53* genes among Japanese patients with mCSPC. Notably, TP53 and biallelic *BRCA2* mutations were significantly associated with poorer CSS, consistent with findings from previous studies in Europe and the United States. Additionally, significant associations were observed between IDC-P and an elevated incidence of genetic alterations, suggesting a potential benefit of early genetic testing in mCSPC patients. A systemic needle biopsy approach is essential to ensure successful genetic testing.

## Supplementary Information

Below is the link to the electronic supplementary material.

Supplementary file1 (XLSX 10 KB)

## Data Availability

The datasets generated during and/or analyzed during the current study are available from the corresponding author on reasonable request.
